# Sheep-Derived *Lactobacillus johnsonii* M5 Enhances Immunity and Antioxidant Capacity, Alleviates Diarrhea, and Improves Intestinal Health in Early-Weaned Lambs

**DOI:** 10.3390/microorganisms13020404

**Published:** 2025-02-13

**Authors:** Zhuo Wang, Yuwei Zhao, Dingkun Fan, Jixian Zhang, Qiyu Diao, Kai Cui

**Affiliations:** Key Laboratory of Feed Biotechnology of the Ministry of Agriculture and Rural Affairs, Institute of Feed Research of Chinese Academy of Agricultural Sciences, Beijing 100081, China; wangzhuo990119@163.com (Z.W.); zhaoyw0010@163.com (Y.Z.); fandingkun1@outlook.com (D.F.); zhangjixian2020@126.com (J.Z.); diaoqiyu@caas.cn (Q.D.)

**Keywords:** *Lactobacillus*, lambs, early weaning, antioxidant, immunity

## Abstract

The early weaning of lambs frequently leads to weakened immunity, impaired intestinal function, and increased susceptibility to intestinal disease. *Lactobacillus* plays a role in regulating immunity, enhancing antioxidant capacity, and maintaining intestinal health. This study aims to isolate a strain of *Lactobacillus* with favorable probiotic properties from sheep feces and investigate its effects on the intestinal health of early-weaned lambs. In this study, the growth characteristics, acid production capacity, bacteriostatic capacity, bile salt tolerance, gastrointestinal fluid tolerance, self-coagulation capacity, and surface hydrophobicity of *Lactobacillus* isolated from sheep feces were analyzed for in vitro probiotic properties. *Lactobacilli* with strong probiotic properties were used for in vivo validation. A total of 72 Hu lambs were allocated into four groups: a ewe-reared group (ER), early-weaning group (EW), low-dose *Lactobacillus* group (LL), and high-dose *Lactobacillus* group (HL). Early weaning was performed in the EW, LL, and HL groups at the age of 28 days. *Lactobacillus johnsonii* M5 (*L. johnsonii* M5), isolated from sheep feces, exhibited strong probiotic properties in vitro. Feeding EW lambs with a low dose of *L. johnsonii* M5 significantly reduced their diarrhea rate (*p* < 0.05). Its supplementation increased the levels of superoxide dismutase (SOD), catalase (CAT), and glutathione peroxidase (GSH-Px) and total antioxidant capacity (T-AOC) in serum and jejunal mucosa and decreased levels of malondialdehyde (MDA) (*p* < 0.05). Compared to the EW group, serum immunoglobulin G (IgG) levels were significantly increased in the LL group (*p* < 0.05). Compared to the EW group, feeding with *L. johnsonii* M5 increased the content of anti-inflammatory cytokines, while reducing the content of pro-inflammatory cytokines in serum and jejunal mucosa (*p* < 0.05). Feeding early-weaned lambs with *L. johnsonii* M5 also decreased jejunal crypt depth and increased occludin and claudin-1 in jejunal mucosa (*p* < 0.05). These findings indicate that feeding early-weaned lambs with *L*. *johnsonii* M5 enhances their immunity and antioxidant capacity, improving intestinal health, and mitigates diarrhea in early-weaned lambs.

## 1. Introduction

The early weaning of lambs is a key practice in large-scale farming. Early weaning improves reproductive efficiency in ewes and facilitates the early feeding of starters, which stimulates rumen development and promotes long-term growth performance in lambs [1,2,3]. However, lambs are highly susceptible to diarrhea after weaning [4], which is often closely associated with changes in nutrient composition and dietary levels [5] and is accompanied by environmental, social, nutritional, and psychological changes [6]. Due to an underdeveloped gastrointestinal tract function and insufficient secretion of digestive enzymes in lambs, weaning stress can cause damage to the intestinal barrier function [7,8]. In addition, weaning stress often leads to imbalances in the antioxidant system and damage to the immune system in lambs [9], which result in increased susceptibility to disease. Ultimately, it causes intestinal inflammation and diarrhea, causing severe economic losses to the sheep industry [10]. Therefore, improving gut health and immunity in weaning-stressed lambs to enhance growth performance and decrease the diarrhea rate is critical to the development of the current sheep industry.

Adding probiotics to the diet has been recognized as a viable nutritional strategy to modulate the immune function and improve gut health [11]. Among the various types of probiotics, lactic acid bacteria are particularly noteworthy. They interact with the host through their own properties, such as activating immune cells, promoting the expression of tight junction protein, and producing metabolites like organic acids and bacteriocins, thereby enhancing intestinal health. *Lactobacillus* represents the most prominent genus of lactic acid bacteria [12], which can modulate immunity, improve intestinal microecological balance, and enhance the integrity of the intestinal barrier [13,14,15]. Feeding *L. johnsonii* to animals improves immune function, antioxidant capacity, and growth performance, effectively alleviating diarrhea caused by weaning stress [16]. A study demonstrated that *Lactobacillus reuteri* and *L. johnsonii*, isolated from calf feces, enhance immune status, antioxidant capacity, intestinal barrier function, and growth performance in weaned piglets [17].

Taking into consideration the aforementioned research, investigating the effects of *Lactobacillus* on growth performance, immunity, antioxidant capacity, and intestinal health in early-weaned lambs is highly valuable. So far, limited research has explored the role of *L. johnsonii* in lambs. We hypothesized that *L. johnsonii* M5, isolated from sheep feces, would alleviate diarrhea caused by weaning stress by improving immunity and intestinal health in early-weaned lambs. Therefore, the objective of this study was to isolate a *Lactobacillus* strain with strong probiotic properties (acid-producing capacity, bacteriostatic capacity, gastrointestinal tolerance, and intestinal adherence) from sheep feces and evaluate its effects on growth performance, diarrhea, antioxidant capacity, immunity, and intestinal health in early-weaned lambs.

## 2. Materials and Methods

### 2.1. Isolation and Identification of Lactobacillus from Sheep Feces

Fresh sheep feces were collected and transported to the laboratory under refrigeration. A 0.5 g fecal sample was diluted in a sterile phosphate-buffered saline (PBS) solution at various ratios. After a 10, 10^2^, and 10^3^ dilution, 100 μL of supernatant was spread onto MRS (de Man, Rogosa, and Sharpe) agar (AOBOX, Beijing, China). The samples were evenly spread using a sterile spreader, and the inoculated plates were incubated anaerobically at 37 °C for 16–24 h in an anaerobic workstation (Defendor AMW 500, Huayue Instrument Co., Ltd., Guangzhou, China). Single, creamy-white colonies were selected and purified through 2–3 rounds of subculturing until a consistent colony morphology was achieved. Gram staining was performed to observe bacterial morphology under oil immersion microscopy (CX31RTSF, Olympus, Tokyo, Japan). The suspected positive, rod-shaped colonies with different morphologies were purified and inoculated in MRS broth for preservation at −80 °C.

Single colonies were cultured in 1 mL MRS broth medium anaerobically at 37 °C for 24 h. Subsequently, 5 µL of bacterial solution and 30 µL of lysate was mixed and incubated at 80 °C for 20 min to obtain bacterial DNA. The bacterial 16S rRNA gene was amplified and sequenced using PCR with universal primers 27F (5′-AGAGTTTGATCCTGGCTCAG-3′) and 1492R (5′-GGTTACCTTGTTACGACTT-3′) [18,19,20]. The PCR conditions included an initial denaturation at 94 °C for 5 min, followed by 40 cycles of denaturation at 94 °C for 30 s, annealing at 56 °C for 30 s, extension at 72 °C for 5 s, and a final extension at 72 °C for 10 min. The reactions consisted of 6 µL of genomic DNA, 1.2 µL of each primer (20 μmol L⁻¹), and 15 µL of 2 × Hieff^®^ PCR Master Mix (With Dye) (yeasen Biotechnology, Shanghai, China), forming a 30 µL reaction mixture. The PCR product was verified on a 1.5% agarose gel, and the final PCR products were sent to Beijing Tsingke Biotech Co., Ltd. for sequencing. Sequence similarity values were identified using the BLAST tool version 2.2.26 of the National Center for Biotechnology Information (NCBI, Rockville, MD, USA). A phylogenetic tree for nucleotide sequences was constructed based on 16S rRNA gene sequences using MEGA software version 7.0 (The Biodesign Institute, Tempe, AZ, USA). Bootstrap analysis was performed with 1000 replicates of the data [21].

### 2.2. Determination of Growth and Acid Production Curves of Lactobacillus

Four single colonies of *Lactobacillus* strains were selected and inoculated into MRS broth medium for anaerobic culturing at 37 °C for 12 h in an anaerobic workstation. These cultures were then transferred into 50 mL MRS broth medium at a 3% inoculation rate and anaerobically cultured at 37 °C for 24 h. Samples were taken every 2 h to measure absorbance at 600 nm (OD_600nm_) using a microplate reader (Agilent, BioTek Synergy H1, Santa Clara, CA, USA) and to determine the pH value using a pH meter (Sartorius, PB-10, Göttingen, Germany).

### 2.3. Bacteriostatic Properties of Lactobacillus (In Vitro)

*Escherichia coli* (*E. coli)* EC87E, *Salmonella* *enteritidis* (*S. enteritidis)* CVCC3377, and *Staphylococcus aureus* (*S. aureus)* ATCC43300 were purified and cultured on Mueller–Hinton (MH) agar plates for three generations. Single colonies were inoculated into Luria-Bertani (LB) broth, cultured for 12 h, and diluted to a concentration of 1 × 10⁶ CFU/mL. The diluted bacterial solution was evenly spread on MH agar plates, and bacteriostatic testing was performed using the Oxford cup method. Four *Lactobacillus* strains were activated and cultured in MRS broth for 24 h, followed by centrifugation at 12,000 rpm for 10 min. Then, 200 μL of fermentation supernatant from each strain was added to an Oxford cup. Each strain was tested in triplicate. The MH agar plates were incubated anaerobically at 37 °C overnight. The bacteriostatic zones were observed, and their diameters were measured using a Vernier caliper (Pro’s Kit, PD-151, Taiwan, China), with data recorded in millimeters.

### 2.4. Bile Tolerance of Lactobacillus (In Vitro)

Bile tolerance tests were performed following the method of Kaushik et al. [22]. The strains were inoculated in MRS liquid medium and cultured at 37 °C for 24 h. The cultures at a stable growth stage were transferred into MRS medium containing 0.3% (*w*/*v*) bile salt at a 5% inoculation size. Samples were collected at 0 and 3 h. The bacterial solutions were serially diluted using sterile PBS, and 100 μL of the appropriate dilution was spread on MRS agar plates. Three plates were inoculated per dilution and incubated anaerobically at 37 °C for 24 h. Plates with colony counts ranging from 30 to 300 were used for viable bacteria enumeration. The experiment was performed in triplicate.

The survival rate (%) = B_3_/B_0_ × 100

where

B_3_ = Number of viable bacteria (*Lactobacillus* strains) treated with bile salt for 3 h.

B_0_ = Number of viable bacteria in the initial solution.

### 2.5. Gastrointestinal Environmental Tolerance Test of Lactobacillus

The tolerance of the strains to artificial gastric fluid and artificial intestinal fluid was assessed as described by Marteau et al. [23] and Suzuki et al. [24]. The methods for culturing *Lactobacillus* and viable bacteria enumeration were consistent with those described above.

The artificial gastric fluid was composed as follows: 3.1 g/L NaCl, 1.1 g/L KCl, 0.15 g/L CaCl_2_-2H_2_O, 0.6 g/L NaHCO_3_.The pH was adjusted to 3.0, pepsin (Solarbio, Beijing, China) was added to the artificial gastric fluid to make the concentration of pepsin 10 g/L, and it was sterile filtrated by filtration with a microporous membrane of 0.22 μL pore size.

For the artificial intestinal fluid, weigh 6.8 g of KH_2_PO_4_ dissolved in 500 mL of water; adjust the pH of the mixture to 6.8 with NaOH; add trypsin (Solarbio, Beijing, China) 10 g/L, with a 0.22 μL pore size for the microporous membrane filtration sterilization.

Survival rate of artificial gastric fluid = (lgN_1_/lgN_0_) × 100%

Survival rate of artificial intestinal fluid = (lgN_2_/lgN_0_) × 100%

where N_0_ is the number of viable bacteria at 0 h (CFU/mL);

N_1_: Number of viable bacteria after 3 h treatment with artificial gastric juice (CFU/mL).

N_2_: Number of viable bacteria after 3 h treatment with artificial intestinal juice (CFU/mL).

### 2.6. Hydrophobicity and Auto-Agglutination Ability Assay of Lactobacillus

Five milliliters of *Lactobacillus* fermentation broth was centrifuged at 8000 rpm for 15 min. The bacteria were washed twice with sterile PBS, and the pellet was resuspended in PBS. The absorbance was adjusted to OD_600nm_ of 0.8 ± 0.05. Three milliliters of *Lactobacillus* culture was mixed with 1 mL of xylene, and the absorbance of the aqueous phase at 600 nm was measured. After 5 min of shaking in a 37 °C water bath, the aqueous phase absorbance was measured again after 20 min of incubation at 37 °C. The hydrophobicity of the *Lactobacillus* was calculated using the following formula:

Hydrophobic force (%) = (A_0_ − A_t_)/A_0_ × 100

where A_0_ and A_t_ are the absorbance values at 0 and 20 min of the mixture of *Lactobacillus* and xylene, respectively.

*Lactobacillus* culture with an absorbance of (OD_600nm_) was adjusted to 0.8 ± 0.05 and incubated at 37 °C for 4 h. The centrifuge tube containing the bacterial suspension was slowly removed from the anaerobic workstation, and 1 mL of the upper layer was taken for measuring the absorbance at 600 nm. The formula for calculating the auto-agglutination rate of *Lactobacillus* is as follows:

Auto-agglutination rate (%) = (B_0_ − B_t_)/B_0_ × 100

where B_0_ and B_t_ are the absorbance values at 0 and 4 h of *Lactobacillus* culture supernatant, respectively.

### 2.7. Safety Evaluation of Lactobacillus

Columbia blood agar base medium (Oxoid, Basingstoke, UK) was prepared with 5% sterile defibrillated sheep blood. Four strains of *Lactobacillus* were inoculated onto blood agar plates using the three-zone streak method. The inverted plates were incubated at 37 °C for 24 h to observe hemolytic activity. A greenish ring around the colony was evaluated as α-hemolysis, while a clear zone around the colony indicated β-hemolysis, also known as complete hemolysis. No changes around the colony were recorded as γ-hemolysis (no hemolysis).

The sensitivity of *Lactobacillus* strains to antibiotics was determined using the Kirby–Bauer test (K-B method). Bacterial cultures were inoculated into fresh MRS broth and incubated anaerobically at 37 °C for 12–16 h. One hundred microliters of activated bacterial suspension was evenly spread on an MRS agar plate with a sterile cotton swab. The plate was left at room temperature for 5–10 min before sterile tweezers were used to place antibiotic discs onto the surface. The plates were incubated at 37 °C for 24 h. The inhibition zones were measured using a Vernier caliper, and results were recorded in millimeters (mm). Drug sensitivity was interpreted based on the Clinical and Laboratory Standards Institute (CLSI) guidelines as sensitive (S), intermediate (I), or resistant (R).

### 2.8. Animals and Experimental Design

The experimental protocol was approved by the Animal Ethics Committee of the Institute of Feed Research of the Chinese Academy of Agricultural Sciences (protocol number: IFR-CAAS20240920). Animal experiments were conducted from August to September 2024 at Linqing Runlin Animal Husbandry Co., Ltd., Liaocheng, China. The *L. johnsonii* M5 used was isolated as described in Section 2.1. The bacterium was inoculated into MRS broth medium at a 3% inoculation size and cultured anaerobically at 37 °C for 24 h using an anaerobic workstation; the cultures were centrifuged to remove the supernatant, and the bacterial pellet was washed with PBS 2~3 times and diluted with PBS to a concentration of 2 × 10⁸ CFU/mL. A total of seventy-two 21-day-old Hu lambs with similar body weights (6.30 ± 0.90 kg) were selected and assigned into four treatment groups (*n* = 18 per group): ER (ewe-reared group, lactation with the mother throughout the experiment); EW (early-weaned group, weaned at 28 days of age and fed starter), LL (low-*Lactobacillus* group, weaned at 28 days of age, fed with 2 × 10⁸ CFU where the *L. johnsonii* M5 suspension was diluted with PBS and starter from 21 days of age), and HL (high-*Lactobacillus* group, weaned at 28 days of age, fed 2 × 10⁹ CFU where the *L. johnsonii* M5 suspension was diluted with PBS and starter from 21 days of age). There were six lambs (*n* = 6) in each pen, and each group had three pens.

### 2.9. Experimental Animals, Diet, and Feeding Management

All ewes were fed twice daily according to the farm’s feeding management schedule. All lambs were nursed by ewes in the same enclosure after birth. Each replicate consisted of six lambs housed together, with free access to starter (provided by Linqing Runlin Animal Husbandry Co., Ltd.) and water. The EW, LL, and HL groups were separated from the ewes at 28 days of the lambs’ life, and all lambs had free access to starter feed and water throughout the whole experiment. The ER group remained suckled by ewes while also consuming starter. Starting on the first day of the trial period (at 21 days of age), *L. johnsonii* M5 was administered orally daily at 9:00 a.m. using syringes containing 2 × 10⁸ CFU/mL. The LL group received 1 mL, while the HL group received 10 mL. The ingredients and analysis of the starter feed are presented in Table 1.

### 2.10. Growth Performance and Diarrhea Rate

The pre-morning feeding weight of all the test lambs was recorded at 21, 28, and 35 days of age. The average daily gain (ADG) was calculated based on the starting and ending weights of each stage. The average daily feed intake (ADFI) of each enclosure was recorded and calculated daily. The feed-to-gain ratio (F/G) was calculated using the ADG and ADFI. The lambs were observed daily after feeding for diarrhea symptoms. Stool scores (1–4 scale) were assigned based on the stool physical state, lambs’ mental condition, and enclosure hygiene. When the stool score was ≥2, the number of days with diarrhea and the number of lambs with diarrhea on each day were recorded. The diarrhea scoring standard was as follows: “firm, well-formed feces” scored 1; “feces soft and resembling pudding” scored 2; “semi-liquid feces” scored 3; and “liquid and splashy feces” scored 4. The diarrhea rate was calculated using the following formula:

Diarrhea rate (%) = (number of lambs with diarrhea × number of days with diarrhea)/(total number of lambs × number of experimental days) × 100.

### 2.11. Blood Sampling, Processing, and Measurement

At 35 days of age, six lambs from each group (*n* = 24) were randomly selected for blood collection via the jugular vein using disposable vacuum sampling vessels. Ten milliliters of blood was collected, left at room temperature for 30 min, and centrifuged at 4000 rpm. The supernatant was collected, transferred to 2 mL centrifuge tubes, and stored at −20 °C. We employed the corresponding colorimetric assay kits (Beijing Jinhai Keyu Biotechnology Development Co., Ltd., Beijing, China) for malondialdehyde (MDA) (Product Number: A003-1), superoxide dismutase (SOD) (Product Number: A001-1), catalase (CAT) (Product Number: A007-2), glutathione peroxidase (GSH-Px) (Product Number: A005) and total antioxidant capacity (T-AOC) (Product Number: A015), immunoglobulin A (IgA) (Product Number: JH-00014), immunoglobulin M (IgM) (Product Number: JH-00015), and immunoglobulin G (IgG) (Product Number: JH-00013). The levels of tumor necrosis factor-α (TNF-α) (Product Number: H502-1-2), interleukin-1β (IL-1β) (Product Number: H002-1-2), interleukin-6 (IL-6) (Product Number: H007-1-1), and interleukin-10 (IL-10) (Product Number: H009-1-1) in serum were evaluated using enzyme-linked immunosorbent assay (ELISA) detection kits (Nanjing Jiancheng Bioengineering Institute, Nanjing, China) and measured with a Multiskan FC microplate reader (Thermo Scientific, Waltham, MA, USA).

### 2.12. Jejunum Sampling, Processing, and Histomorphology Analysis

On day 35 of age, after blood sampling, six lambs from each group (*n* = 24) were sacrificed to collect intestinal samples. The middle part of the jejunum was sampled, washed with saline to remove chyme, and fixed in 4% paraformaldehyde for intestinal morphology analysis. Tissues were dehydrated with ethanol and toluene, embedded in paraffin, sectioned, and stained with hematoxylin and eosin (H&E). Villus height, crypt depth, and the villus height-to-crypt depth ratio (V/C) were measured using Digital Pathology Viewer software (v2.0.4.0104, Shenzhen Shenggiang Technology Co., Ltd., Shenzhen, China) [25]. The intestinal ring was cut open with surgical scissors, and the mucosal tissue on the inner side of the intestinal segment was carefully scraped using a disposable glass slide. The tissue was placed in a 2 mL freezing tube, flash-frozen in liquid nitrogen, and stored at −80 °C for further analysis. The jejunum mucosa (1 g) was homogenized and mixed with 9 mL of normal saline. The method for detecting levels of MDA, SOD, CAT, GSH-Px, T-AOC, TNF-α, IL-1β, IL-6, and IL-10 in the jejunum mucosa were the same as Section 2.11. The levels of IL-4 (Product Number: H005-1-1), IL-17 (Product Number: H014-1), and tight junction proteins ZO-1 (Product Number: H373-1-1), claudin-1 (Product Number: H632), and occludin (Product Number: H369-1-1) in the jejunum mucosa were evaluated using enzyme-linked immunosorbent assay (ELISA) detection kits (Nanjing Jiancheng Bioengineering Institute, Nanjing, China) and measured with a Multiskan FC microplate reader (Thermo Scientific, Waltham, MA, USA).

### 2.13. Statistical Analysis

The results were analyzed using a one-way analysis of variance (ANOVA) with SPSS version 20.0 (IBM Corp, Armonk, NY, USA). Duncan’s multiple range test was used to determine significant differences among groups at *p* < 0.05. Visualizations were generated using GraphPad Prism version 8.0.2 (GraphPad Software, Dotmatics, Boston, MA, USA).

## 3. Results

### 3.1. Isolation and Identification of Lactobacillus

**Figure 1:**
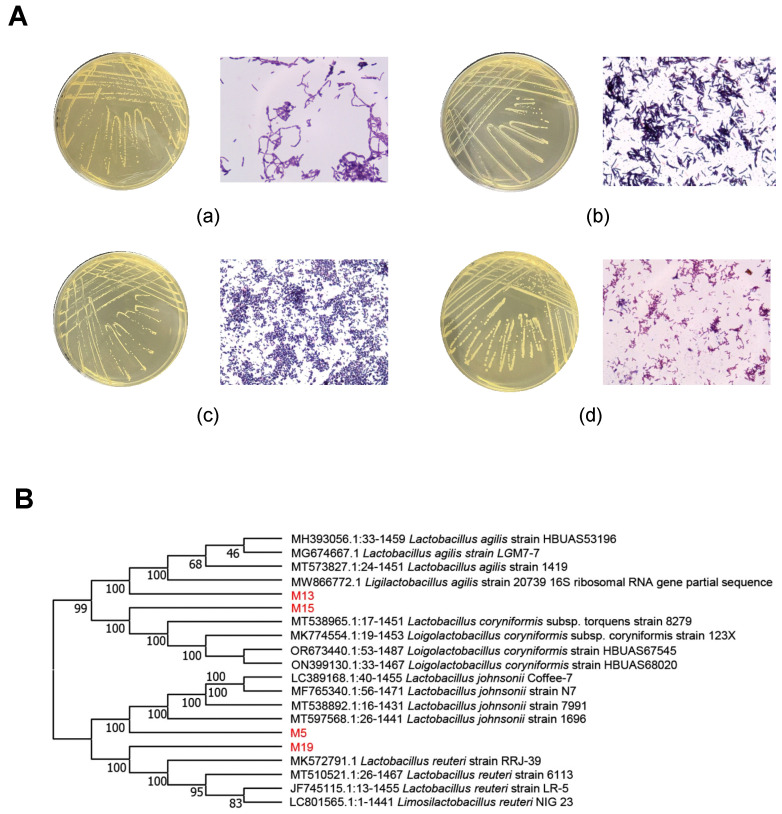
(**A**) Colony morphology of *Lactobacillus* strains isolated from sheep feces on MRS agar medium and the microscopy results of Gram staining. (**a**) Strain M5; (**b**) strain M13; (**c**) strain M15; (**d**) strain M19. (**B**) Phylogenetic tree derived from 16S rRNA gene sequence of *Lactobacillus* isolated from sheep feces.

Single colonies with milky white, slightly raised, and smooth surfaces were selected on MRS solid medium. The isolates were purified and subjected to Gram staining for preliminary identification, revealing four morphologically distinct strains that stained positively as rod-shaped bacteria (Figure 1A). Species and genus identification of the four *Lactobacillus* strains were determined by comparing sequencing results with NCBI data. The phylogenetic tree was constructed using MEGA7.0, and the results are shown in Figure 1B. Strains were identified as follows: M5, *Lactobacillus johnsonii*; M13, *Lactobacillus agilis*; M15, *Loigolactobacillus coryniformis*; and M19, *Lactobacillus reuteri*.

### 3.2. Growth Curve and Acid Production Curve

**Figure 2:**
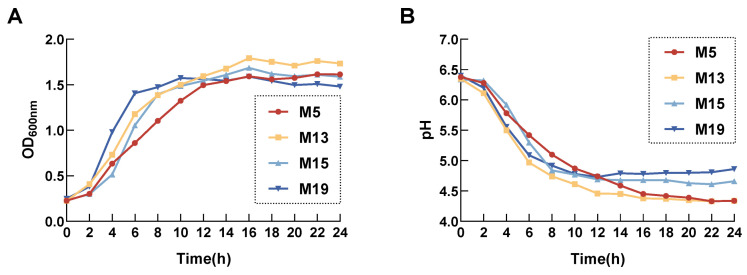
(**A**) Growth curves of the four *Lactobacillus* strains isolated from sheep feces after 24 h. (**B**) Acid production curves of the four *Lactobacillus* strains isolated from sheep feces after 24 h.

The growth curves of the four isolates are presented in Figure 2. Strain M5 grew slowly during the first 2 h, entered the logarithmic stage between 2 and 12 h, achieved the fastest growth rate, and plateaued after 12 h. Strains M13 and M15 entered the logarithmic stage between 2 and 8 h, reaching their fastest growth rates, and plateaued after 16 h and 8 h, respectively. Strain M19 grew fastest between 4 and 6 h, entering the logarithmic phase, and plateaued after 6 h with a slower growth rate. The pH of strains M5 and M13 decreased to below 4.5 after 24 h of growth, while the pH of strains M15 and M19 decreased to below 5 during the same period.

### 3.3. In Vitro Bacteriostatic Performance

**Figure 3:**
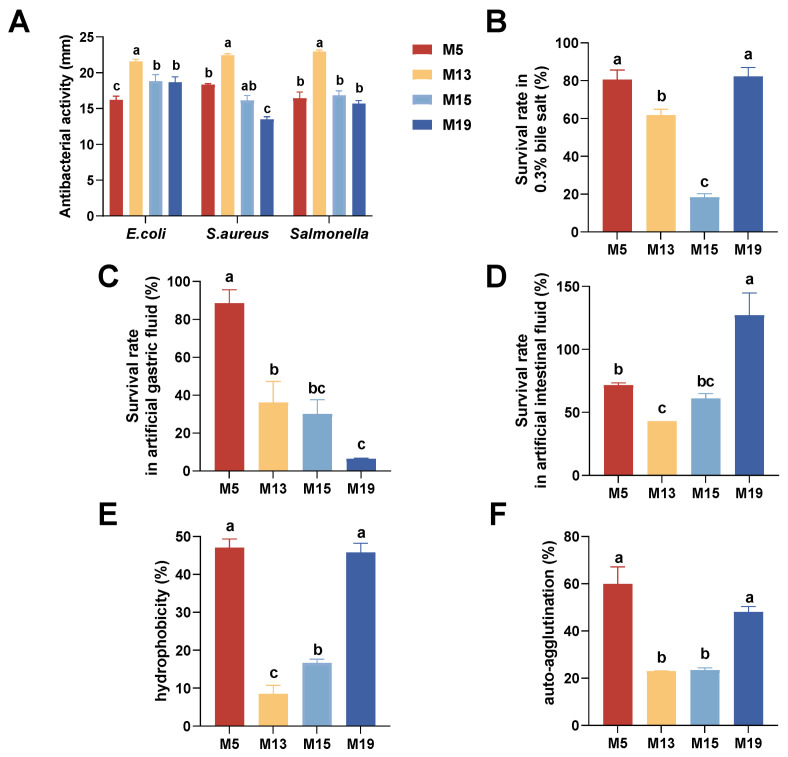
(**A**) Inhibitory zone diameters of four strains of *Lactobacillus* isolated from sheep feces against *E. coli*, *S. aureus,* and *E. enteritidis*. Survival of the four strains of *Lactobacillus* in (**B**) 0.3% bile salts, (**C**) artificial gastric juice at pH 3.0, and (**D**) artificial intestinal juice at pH 6.8. (**E**) Hydrophobicity and (**F**) auto-agglutination rate of the four *Lactobacillus* strains. The same superscript letters indicate no significant difference (*p* > 0.05), while different letters indicate significant differences (*p* < 0.05).

The four isolates exhibited excellent inhibitory effects on *E. coli*, *S. aureus,* and *S. enteritidis* (Figure 3A). The inhibition zones for *E. coli* were as follows (in descending order): M13 (21.60 ± 0.29 mm), M15 (18.81 ± 0.93 mm), M19 (18.66 ± 0.76 mm), and M5 (16.21 ± 0.51 mm). The inhibition zones for *S. aureus* were M13 (22.44 ± 0.21 mm), M5 (18.33 ± 0.13 mm), M15 (16.13 ± 0.69 mm), and M19 (13.51 ± 0.35 mm). The inhibition zones for *E. Salmonella* were M13 (22.96 ± 0.23 mm), M15 (16.84 ± 0.62 mm), M5 (16.43 ± 0.88 mm), and M19 (15.69 ± 0.43 mm). Among the isolates, *Lactobacillus agilis* M13 demonstrated inhibition zones exceeding 20 mm for all three pathogens.

### 3.4. Bile Salt Resistance

Survival rates of the four *Lactobacillus* strains differed significantly (*p* < 0.05) after 3 h of incubation in MRS medium containing 0.3% bile salts (Figure 3B). Strains M5 and M19 were the most bile salt-tolerant, with survival rates of 82.31% and 80.64%, respectively. Strains M13 and M15 exhibited survival rates of 61.85% and 18.38%, respectively.

### 3.5. Resistance to Artificial Intestinal and Gastric Juices

After 3 h of incubation in artificial gastric juice at pH 3.0, the survival rates of the four *Lactobacillus* isolates differed significantly (*p* < 0.05) (Figure 3C). The survival rate of *L. johnsonii* M5 was 88.63%, indicating strong tolerance to artificial gastric juice. The survival rates of *Lactobacillus agilis* M13 and *Loigolactobacillus coryniformis* M15 were 36.10% and 30.06%, respectively. *Lactobacillus reuteri* M19 demonstrated the weakest resistance, with a survival rate of 6.54%.

After 3 h of incubation in artificial intestinal fluid at pH 6.8, the survival rates of the isolates also showed significant differences (*p* < 0.05) (Figure 3D). *Lactobacillus reuteri* M19 exhibited the strongest tolerance in artificial intestinal fluid, with a survival rate exceeding 100%. The survival rate of *L. johnsonii* M5 was 71.58%, while those of *Loigolactobacillus coryniformis* M15 and *Lactobacillus agilis* M13 were 61.15% and 43.08%, respectively.

### 3.6. Hydrophobic and Auto-Agglutination Tests

The hydrophobicity results of the isolated strains are shown in Figure 3E. Except for strain M13, the other three strains displayed hydrophobic interactions greater than 10%. The strain with the highest hydrophobicity was *L. johnsonii* M5 (47.07%), followed by *Lactobacillus reuteri* M19 (45.81%). *Loigolactobacillus coryniformis* M15 and *Lactobacillus agilis* M13 had hydrophobicity values of 16.63% and 8.58%, respectively.

The auto-agglutination results are presented in Figure 3F. *L. johnsonii* M5 had the highest rate (59.9%), followed by *Lactobacillus reuteri* M19 (48.13%). *Loigolactobacillus coryniformis* M15 and *Lactobacillus agilis* M13 had rates of 23.42% and 22.94%, respectively.

The same letter indicates no significant difference (*p* > 0.05), while different letters indicate significant differences (*p* < 0.05).

### 3.7. Hemolysis and Antibiotic Resistance Tests

**Figure 4:**
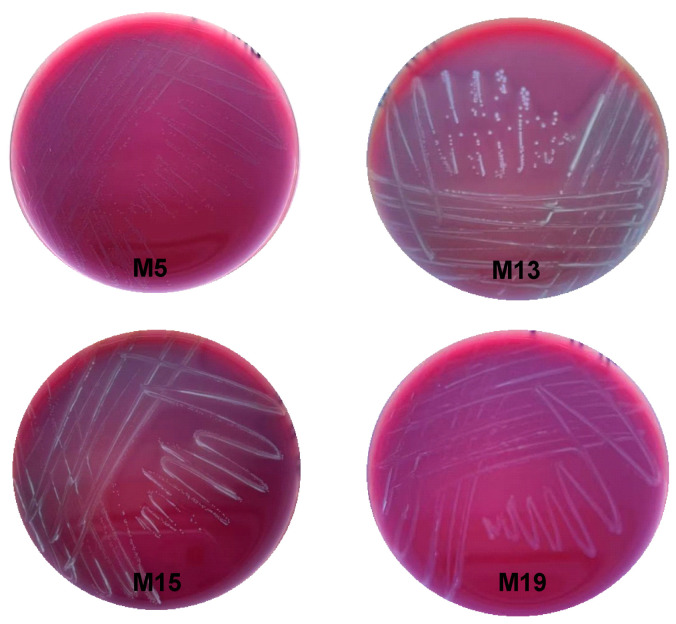
Hemolytic results of the four *Lactobacillus* strains isolated from sheep feces.

To evaluate the safety of the isolates, their hemolysis and antibiotic sensitivities were then tested. From Figure 4, we can see that the four *Lactobacilli* exhibit gamma-hemolysis, indicating no hemolytic activity. From Table 2, we can see that all strains were sensitive or moderately sensitive to Chloramphenicol, and all showed resistance to Amikacin.

### 3.8. Effect of L. johnsonii M5 on the Growth Performance of Weaned Lambs

Table 3 presents the effects of *L. johnsonii* M5 on the growth performance of weaned lambs. Throughout the experiment, the BW and ADG did not significantly differ among groups (*p* > 0.05). At 21 to 28 days of age, the ADFI and F/G were not significantly different between the four groups (*p >* 0.05). At 28 to 35 days of age, the ADFI and F/G were significantly lower in the ER group compared to the other three groups (*p <* 0.05).

### 3.9. Effect of L. johnsonii M5 on Diarrhea in Weaned Lambs

As shown in Table 4, diarrhea rates among all groups at 21–28 days of age were not significantly different (*p* > 0.05). At 28 to 35 days of age, the diarrhea rates in the ER and HL groups were significantly lower than in the EW group (*p* < 0.05). There was no significant difference in the rate of diarrhea between the HL group and the ER and EW groups (*p* > 0.05).

### 3.10. Effect of L. johnsonii M5 on Antioxidant Indices of Weaned Lambs

**Figure 5:**
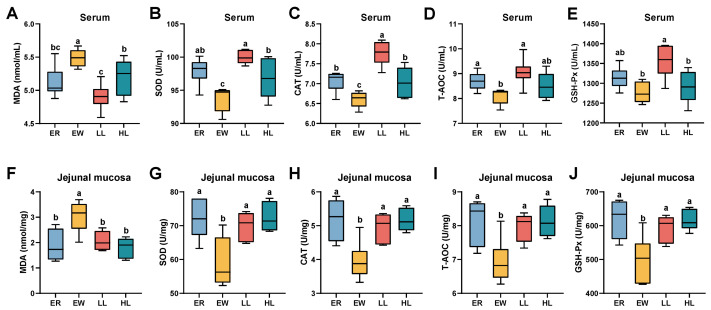
Effects of different doses of *L. johnsonii* M5 on antioxidant indices in weaned lambs (*n* = 6). MDA, malondialdehyde; SOD, superoxide dismutase; CAT, catalase; GSH-Px, peroxidase, glutathione; T-AOC, total antioxidant capacity. In a box plot, the same letters above the box indicate no significant difference (*p* > 0.05), while different letters indicate significant differences (*p* < 0.05). The levels of (**A**) MDA, (**B**) SOD, (**C**) CAT, (**D**) T-AOC and (**E**) GSH-Px in serum of lambs in each group. The levels of (**F**) MDA, (**G**) SOD, (**H**) CAT, (**I**) T-AOC and (**J**) GSH-Px in jejunal mucosa in each group.

As shown in Figure 5A, the serum MDA content was significantly lower in the LL group compared to the HL group and significantly lower in the HL group than in the EW group (*p* < 0.05). The SOD and CAT levels were significantly higher in the LL group than in the HL group and significantly higher in the HL group than in the EW group (Figure 5B,C; *p* < 0.05). Compared with the EW group, T-AOC and GSH-Px levels were significantly higher in the LL group (Figure 5D,E; *p* < 0.05).

As shown in Figure 5F, jejunal MDA levels were significantly lower in the ER, LL, and HL groups compared to the EW group (*p* < 0.05). Conversely, SOD, CAT, T-AOC, and GSH-Px levels were significantly higher in the ER, LL, and HL groups than in the EW group (Figure 5G–J; *p* < 0.05).

### 3.11. Effect of L. johnsonii M5 on Immune Parameters in Weaned Lambs

**Figure 6:**
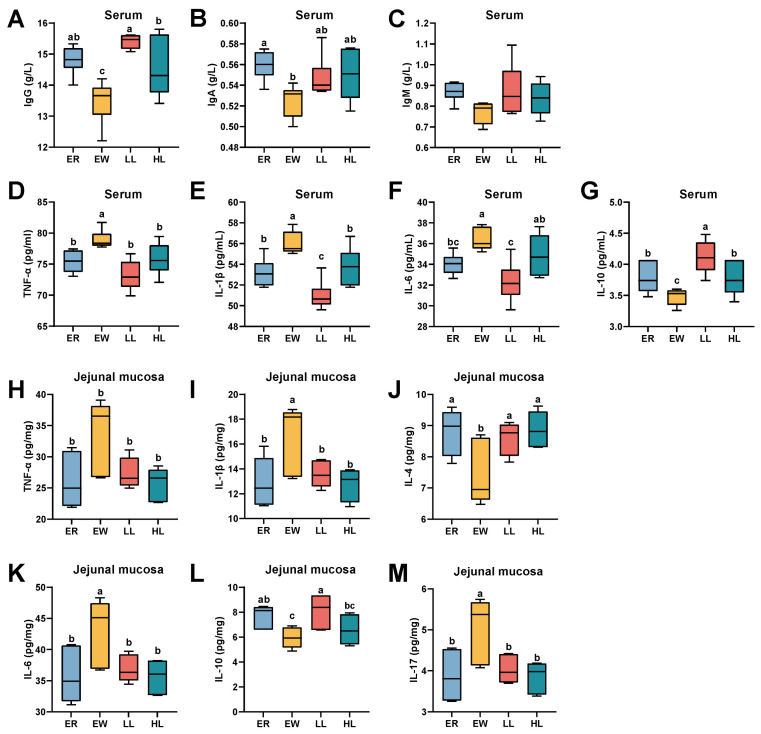
Effects of different doses of *L. johnsonii* M5 on serum immune parameters of weaned lambs (*n* = 6). IgA, immunoglobulin A; IgG, immunoglobulin G; IgM, immunoglobulin M; TNF-α, tumor necrosis factor-α; IL-1β, interleukin-1β; IL-6, interleukin-6; IL-10, interleukin-10. In a box plot, the same letters above the box indicate no significant difference (*p* > 0.05), while different letters indicate significant differences (*p* < 0.05). The levels of (**A**) IgG, (**B**) IgA, (**C**) IgM, (**D**) TNF-α, (**E**) IL-1β, (**F**) IL-6 and (**G**) IL-10 in serum of lambs in each group. The levels of (**H**) TNF-α, (**I**) IL-1β, (**J**) IL-4, (**K**) IL-6, (**L**) IL-10, (**M**) IL-17 in jejunal mucasa of lambs in each group.

As shown in Figure 6A, IgG levels in serum were significantly higher in the LL, ER and HL groups than in the EW group (*p* < 0.05). IgG levels were significantly higher in the LL group than in the HL group (*p* < 0.05). And there was no significant difference between the IgG levels in the ER group and those in both the LL and HL groups (*p >* 0.05). However, IgA and IgM levels in the LL and HL groups did not differ significantly from those in the EW group (Figure 6B,C; *p* > 0.05). Serum IgA levels were significantly higher in the ER group than in the EW group (Figure 6B; *p <* 0.05). As shown in Figure 6D,E, pro-inflammatory cytokines TNF-α and IL-1β were significantly lower in the ER, LL, and HL groups compared to the ER group (*p* < 0.05). In addition, the serum levels of IL-1β in the LL group were significantly lower than those in the ER and HL groups (Figure 6E; *p <* 0.05). As shown in Figure 6F, serum IL-6 levels were significantly lower in the ER and LL groups than in the EW group (*p* < 0.05), and IL-6 levels were significantly lower in the LL group than in the HL group (*p* < 0.05), and there were no significant differences between the HL group and either the ER or EW groups (*p >* 0.05). The anti-inflammatory cytokine IL-10 level was significantly higher in the LL, HL, and ER groups compared to the EW group, with IL-10 in the LL group significantly exceeding levels in the ER and HL groups (Figure 6G; *p* < 0.05).

As shown in Figure 6H–M, pro-inflammatory cytokines TNF-α, IL-1β, IL-6, and IL-17 levels were significantly lower in the ER, LL, and HL groups compared to the EW group (*p* < 0.05). Conversely, the anti-inflammatory cytokine IL-4 level in the EW group was significantly lower than in the ER, LL, and HL groups. Additionally, IL-10 levels were significantly higher in the LL group compared to the EW group (*p* < 0.05).

### 3.12. Effect of L. johnsonii M5 on Jejunal Histomorphology in Weaned Lambs

**Figure 7:**
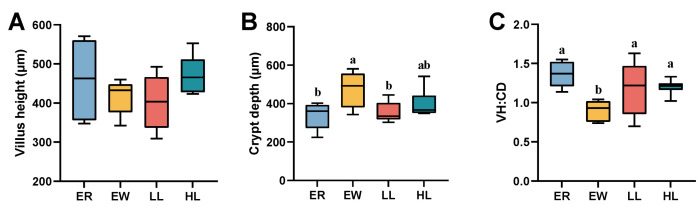
Effects of different doses of *L. johnsonii* M5 on jejunal histomorphology of weaned lambs (*n* = 6). VH:CD, villus height/crypt depth. In a box plot, the same letters above the box indicate no significant difference (*p* > 0.05), while different letters indicate significant differences (*p* < 0.05). The (**A**) villus height, (**B**) crypt depth and (**C**) VH:CH of the jejunum in each group of lambs.

As shown in Figure 6A, there was no significant difference in villus height between the four groups (*p* > 0.05). Crypt depth was significantly lower in the ER and LL groups than in the EW group (Figure 7B; *p* < 0.05). VH:CD was significant higher in the ER, LL, and HL groups than the EW group (Figure 7C; *p* < 0.05).

### 3.13. Effect of L. johnsonii M5 on Tight Junction Protein in Weaned Lambs

**Figure 8:**
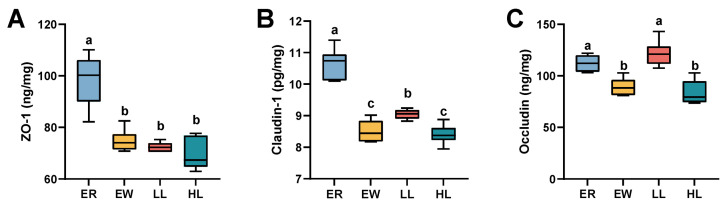
Effects of different doses of *L. johnsonii* M5 on tight junction protein of weaned lambs (*n* = 6). ZO-1, Zonula Occludens-1. In a box plot, the same letters above the box indicate no significant difference (*p* > 0.05), while different letters indicate significant differences (*p* < 0.05). The levels of (**A**) ZO-1, (**B**) claudin-1, (**C**) occludin in jejunal mucasa of lambs in each group.

As shown in Figure 8A, ZO-1 content in the jejunal mucosa of the EW, LL, and HL groups was significantly lower than that of the ER group (*p* < 0.05). As shown in Figure 8B, the levels of claudin-1 in the jejunal mucosa were significantly lower in the EW, LL, and HL groups than in the ER group (*p* < 0.05), and the levels of claudin-1 were significantly lower in the EW and HL groups than in the LL group (*p* < 0.05). The occludin content in the jejunal mucosa was significantly higher in the ER and LL groups than in the EW and HL groups (Figure 8C; *p* < 0.05).

## 4. Discussion

The rationale for probiotics use in animals for their potential health benefits depends on their ability to survive through the gastrointestinal tract (GIT) and interact with or adhere to and colonize the host [26]. Various compounds, such as gastric acid, bile salts, and enzymes, contained in the GIT of ruminants are required for the digestion and absorption of nutrients [27]. In this study, strains M5 and M19 exhibited the highest survival rates in both simulated gastrointestinal environments and bile salt conditions. Liu et al. [23] found in their study that various *Lactobacillus* strains extracted from the feces of healthy young individuals exhibited survival rates of over 50% after incubation in artificial intestinal fluid for 3 h. Cheon et al. [28] reported that the highest survival rate among seven strains of *Lactobacillus* isolated from the dung and milk of Tibetan yaks, which showed the best tolerance to artificial gastric juices, was 57.4%. Additionally, the colonization of *Lactobacillus* in the intestine depends not only on the GIT environment but also on the adhesion ability of probiotics. Aggregation is a key feature of probiotics in forming biofilms, and both aggregation and the hydrophobicity of the cell surface are crucial for promoting the contact between microorganisms and the intestinal wall [29]. Strains with a stronger adhesion ability exhibit greater hydrophobicity and self-agglutination [30]. According to the criteria for bacterial hydrophobicity classification established by Tyfa et al. [31], M5 and M19 are moderately hydrophobic, while M13 and M15 are hydrophilic. Lambs are highly susceptible to pathogenic bacteria after weaning, when the intestinal mucosal barrier is damaged, and immunity is reduced, often leading to intestinal inflammation and diarrhea. Studies have shown that *Lactobacillus* exhibits broad bacteriostatic properties [32,33]. Organic acids, bacteriocins, and other metabolites produced during lactic acid bacteria metabolism play an antibacterial role by altering the permeability of pathogenic bacteria. These metabolites inhibit the growth of pathogens within the host. The inhibition zones of strains M5, M13, and M15 against *E. coli, S. aureus*, and *S. enteritidis* all ranged from 15 mm to 25 mm. Research by Yang et al. indicated that the inhibition zones of *Lactobacillus plantarum* 4-10 against *E. coli* and *S. aureus* ranged between 15 mm and 22 mm [34]. In this research, all four strains demonstrated strong reproductive and acid-producing capacities. With the increased misuse of antibiotics, drug-resistance genes are now considered emerging environmental pollutants [35]. The four *Lactobacillus* strains in this study were all sensitive to multiple antibiotics. None exhibited α-hemolytic or β-hemolytic activity. Thus, all four strains were considered safe regarding antibiotic resistance and hemolysis. In summary, *L. johnsonii* M5 emerged as the best-performing strain in this study. Therefore, this strain was selected for further animal testing to evaluate its effects on weaned lambs.

If lambs are weaned early, they may not adapt to the environment, and their nutrient intake will undergo a sudden change, leading to decreased immunity, a damaged intestinal structure, intestinal barrier dysfunction [36], decreased feed intake, growth retardation [1], and an increased diarrhea rate [37]. Post-weaning diarrhea in lambs is a multifactorial syndrome. Changes in dietary composition and imbalances in intestinal flora during weaning may damage the intestinal barrier, leading to inflammation and diarrhea [38]. Harmful bacterial proliferation triggers inflammation, producing high levels of reactive oxygen and nitrogen species, which result in oxidative stress [39,40]. Oxidative stress occurs when animals experience diarrhea [41]. Antioxidant enzymes, such as SOD, CAT, and GSH-Px, are essential for maintaining redox balance and mitigating oxidative stress damage [42,43,44]. MDA, a final product of lipid peroxidation, serves as a marker of oxidative stress. Studies report that diarrhea often correlates with decreased MDA levels, consistent with this study’s findings [41,45]. In this study, serum and intestinal mucosa MDA levels increased, while antioxidant enzymes and total antioxidant capacity decreased during weaning. Supplementation with *L. johnsonii* M5 increased the levels of antioxidant enzymes, enhancing the body’s antioxidant capacity, thereby reducing the production of MDA generated from lipid peroxidation. Yang et al. [46] reported that dietary supplementation with *Lactobacillus reuteri* could reduce the activities of plasma MDA content in piglets. Studies confirm that *Lactobacillus* regulates the antioxidant system and alleviates oxidative stress by enhancing the activity of enzymes like SOD, CAT, and GSH-Px [18,47,48]. These findings are consistent with the results of our study, which demonstrate that feeding *L. johnsonii* M5 enhances antioxidant capacity and mitigates oxidative stress in weaned lambs, with a more pronounced effect observed at a dosage of 2 × 10⁸ CFU per day.

Diarrhea often occurs with a weakened immune function and intestinal inflammation [18,49]. In early-weaned lambs, a reduced maternal immunoglobulin supply and a weakened immune system increase intestinal tract susceptibility to pathogens, causing diarrhea [50]. Immunoglobulins and cytokines serve as key indicators of immune function and inflammation levels in animals [51]. In this study, feeding with *Lactobacillus* significantly elevated serum IgG levels in weaned lambs, and the levels of IgA and IgM showed an upward trend. Consistent with these findings, Wang et al. [52] reported that *L. johnsonii* BS15 increased serum IgA and IgG concentrations in broiler chickens, enhancing their immunity. The observed effects in this study likely stem from *L. johnsonii* M5, which enhances immunity and accelerates immune response frequency, thereby boosting immunoglobulin content. Cytokines, small proteins produced by immune and some non-immune cells, are critical for activating the immune response [53]. Probiotics have been shown to activate immune responses by modulating cytokine expression and reducing inflammation [54,55]. In this study, weaning stress significantly decreased pro-inflammatory cytokines (IL-6, IL-1β, TNF-α, IL-17) in serum and jejunal mucosa, while increasing anti-inflammatory cytokines (IL-10, IL-4). Similar to this study, probiotics such as *Lactobacillus rhamnosus* can significantly increase the production of anti-inflammatory cytokine IL-10 by stimulating T helper 2 cell (Th2) lymphocytes and macrophages, while reducing the secretion of the major pro-inflammatory cytokines IL-1β and IL-6 by 70% and 80%, respectively [56]. The study by Vinderola et al. [57] demonstrated that *Lactobacillus casei* and *Lactobacillus helveticus* can increase IL-6 levels in a toll-like receptor (TLR2)-dependent manner, triggering B cell expansion and subsequent immunoglobulin release. Feeding with *Lactobacillus* significantly improved these imbalances, enhancing anti-inflammatory capacity and reducing inflammation, thereby alleviating diarrhea.

Intestinal morphology plays a crucial role in the digestion, absorption, and utilization of nutrients [58]. The villus height and crypt depth of the small intestine are key indicators that determine the development and renewal of intestinal epithelial cells [59]. Weaning stress can impair intestinal mucosal function, leading to a reduction in villus height and an increase in crypt depth [39]. In our study, supplementation with *L. johnsonii* M5 significantly reduced crypt depth and increased the villus height to crypt depth ratio. Li et al. [17] reported that supplementation with *Lactobacillus reuteri* significantly increased the villus height and reduced the crypt depth in the small intestine of weaned calves. Tight junctions mainly include occludin, claudin-1, and ZO-1 proteins, whose structures are closely related to the integrity of the intestinal barrier [60]. Studies have shown that *Lactobacillus paracasei* can increase the expression levels of tight junction proteins by inhibiting the Nuclear Factor-κB signaling pathway [61]. When the feeding dose of *L. johnsonii* M5 was 2 × 10^8^ CFU/d, the levels of claudin-1 and occludin in the jejunum significantly increased compared to the EW group.

In general, *Lactobacillus* improves growth performance [62], intestinal health [63], and immune capacity [32] in livestock and poultry, but experimental outcomes vary. In this study, there were no significant differences in ADG or BW among the four groups of animals during the two-week trial. This may be due to the short feeding duration, which did not affect body weight. Lambs in the ER group consumed significantly less starter after weaning than those in the other groups, as they were breastfed in addition to starter. Similarly, He et al. [64] reported no significant body weight differences at 14 days before feeding *Lactobacillus plantarum* to weaned piglets. At 28 days, body weight in the *Lactobacillus* group increased significantly in their study. The effects of *Lactobacillus* on livestock and poultry growth performance differ depending on the animal species, growth environment, and strain properties. Consistent with the present study, Zuniga et al. [65] found no significant effects on BW or ADG in weaned piglets fed *Lactobacillus* and *Bifidobacterium*. However, other studies reported that BW, ADG, and ADFI increased significantly, while the F/G decreased when weaned piglets were fed inactivated *Lactobacillus rhamnosus* [66]. Our study also found a significant increase in diarrhea rate after early weaning. Feeding *L. johnsonii* M5 at 2 × 10⁸ CFU/d significantly reduced diarrhea rates. Yin et al. [16] observed similar effects in weaned piglets fed *Lactobacillus johnsonii*. Based on the aforementioned results, we can conclude that feeding *L. johnsonii* M5 to early-weaned lambs did not significantly enhance growth performance in the short term. However, during the trial period, its supplementation enhanced intestinal health and immune function, which may indirectly improve growth performance through long-term supplementation. In addition, we can conclude that supplementing with a low dose of *L. johnsonii* M5 is more beneficial for improving immunity, antioxidant capacity, intestinal morphology, and the expression of tight junction proteins in early-weaned lambs. This may be attributed to the beneficial effects of metabolites produced by *Lactobacillus*, such as lactic acid and short-chain fatty acids (e.g., acetate, propionate, and butyrate), at low doses. However, a high dose may lead to an excessively acidic environment, potentially disrupting the intestinal pH. Additionally, a high dose could put excessive stress on intestinal epithelial cells, impairing the barrier function instead [67].

## 5. Conclusions

In this study, four *Lactobacillus* strains with varying morphologies were isolated from sheep feces, and *L. johnsonii* M5 was identified as having the strongest probiotic properties based on an in vitro characterization. In vivo experiments showed that *L. johnsonii* M5 increased antioxidant capacity and immunity, improved intestinal health and reduced diarrhea in early-weaned lambs, and the dose with the best improvement effect was 2 × 10⁸ CFU/d.

## Figures and Tables

**Table 1:** Composition and nutrient levels of the starter feed (dry matter base, %).

Ingredient Composition	Content	Nutrient Levels	Content (Dry Matter Base %)
Corn	56.8	DE (MJ/kg)	16.68
Soybean meal	23.0	CP	18.02
Corn germ meal	10.0	EE	3.68
Bran	5.0	NDF	22.03
Baking soda	0.5	ADF	7.37
Stone powder	2.0	Coarse Ash	11.00
Salt	0.5	Ca	0.92
Calcium hydrogen phosphate	1	TP	0.12
Additive premix	1		
Mold inhibitor	0.2		

DE, digestive energy; CP, crude protein; EE, ether extract; NDF, neutral detergent fiber; ADF, acid detergent fiber; Ca, calcium; TP, total phosphorus.

**Table 2:** Drug resistance of the four *Lactobacillus* strains isolated from sheep feces.

Antibiotics	Inhibition Zone Diameter (mm) and Sensitivity Type			
	M5	M13	M15	M19
Chloramphenicol (30 μg)	32.81 (S)	16.46 (I)	33.24 (S)	19.04 (S)
Amoxicillin (25 μg)	0 (R)	15.70 (I)	24.92 (S)	27.38 (S)
Norfloxacin (10 μg)	0 (R)	11.91 (R)	15.56 (I)	0 (R)
Tetracycline (30 μg)	19.37 (S)	21.37 (S)	0 (R)	21.27 (S)
Neomycin (30 μg)	0 (R)	8.77 (R)	15.23 (S)	10.76 (I)
Gentamicin (10 μg)	0 (R)	0 (R)	16.0 (S)	0 (R)
Amikacin (30 μg)	0 (R)	0 (R)	13.16 (R)	14 (R)
Ceftriaxone (30 μg)	0 (R)	20.97 (S)	23.61 (S)	25.93 (S)
Cefazolin (30 μg)	0 (R)	23.33 (S)	26.55 (S)	25.83 (S)
Cephalexin (30 μg)	16.99 (S)	13.39 (R)	17.70 (I)	0 (R)
Piperacillin (100 μg)	0 (R)	28.65 (S)	13.13 (R)	30.03 (S)
Oxacillin (1 μg)	20.27 (S)	11.85 (R)	14.42 (R)	16.78 (S)

Note: S, sensitive; I, intermediate; R, insensitive.

**Table 3:** Effect of different doses of *L. johnsonii* M5 on growth performance of weaned lambs.

Items	Group ^1^				SEM	*p*-Value
	ER	EW	LL	HL		
BW (kg)						
21 d	6.11	6.15	6.55	6.35	0.11	0.440
28 d	7.28	7.47	7.89	7.47	0.15	0.498
35 d	8.37	8.43	9.15	8.32	0.17	0.236
ADG (g/d)						
21–28 d	166.86	193.81	190.28	160.23	9.42	0.514
28–35 d	156.75	146.67	179.76	127.38	8.21	0.137
ADFI (g/d)						
21–28 d	88.89	138.10	131.35	151.59	14.97	0.545
28–35 d	147.22 ^b^	295.71 ^a^	324.21 ^a^	286.11 ^a^	23.93	0.009
F/G						
21–28 d	0.54	0.77	0.64	1.03	0.09	0.263
28–35 d	0.95 ^b^	2.27 ^a^	1.78 ^a^	2.15 ^a^	0.20	0.003

Note: BW, body weight; ADG, average daily gain; ADFI, average daily feed intake; F/G, feed-to-gain ratio = ADFI/ADG. ^1^ ER, ewe-reared group; EW, early weaning group; LL, low-dose supplement group; HL, high-dose supplement group. The same superscript letters indicate no significant difference (*p* > 0.05), while different letters indicate significant differences (*p* < 0.05).

**Table 4:** Effect of different doses of *L. johnsonii* M5 on diarrhea of weaned lambs.

Diarrhea (%)	Group				SEM	*p*-Value
	ER	EW	LL	HL		
21–28 d	27.78	27.78	20.37	18.06	3.29	0.725
28–35 d	40.74 ^b^	55.56 ^a^	42.08 ^b^	46.30 ^ab^	2.15	0.028

ER, ewe-reared group; EW, early weaning group; LL, low-dose supplement group; HL, high-dose supplement group. The same superscript letters indicate no significant difference (*p* > 0.05), while different letters indicate significant differences (*p* < 0.05).

## Data Availability

The original contributions presented in this study are included in the article. Further inquiries can be directed to the corresponding author.
